# The Valorization of Rapeseed Meal as Hydrolyzed and Lyophilized Extract to Improve the Antioxidant Properties of Refined Rapeseed Oil During Frying and Fried French Fries

**DOI:** 10.3390/foods14091444

**Published:** 2025-04-22

**Authors:** Dobrochna Rabiej-Kozioł, Aleksandra Szydłowska-Czerniak

**Affiliations:** Department of Analytical Chemistry and Applied Spectroscopy, Faculty of Chemistry, Nicolaus Copernicus University in Toruń, Gagarina 7, 87-100 Toruń, Poland; d.rabiej@umk.pl

**Keywords:** oil by-products, hydrolysis, *Brassica napus* seed oil, thermo-degradation process, oxidation parameters, polar fraction, antioxidant activity, food matrix

## Abstract

In the present study, methanolic extracts from rapeseed meal, an oil industry by-product, were treated with alkaline hydrolysis, acid hydrolysis, and lyophilization to enhance their antioxidant features. Antioxidant activity (AA) of the prepared rapeseed meal extracts was determined using three modified spectrophotometric methods: 2,2′-azino-bis(3-ethylbenzothiazoline-6-sulfonic acid) (ABTS), 2,2-diphenyl-1-picrylhydrazyl (DPPH), and ferric reducing antioxidant power (FRAP) methods. The effect of acid-hydrolyzed and lyophilized rapeseed meal extract (HLRME) at 200 ppm on the antioxidant properties of refined rapeseed oil heating at 180 °C for 24 h and French fries fried in it was estimated. Moreover, the total phenolic content (TPC) in rapeseed meal extracts, enriched rapeseed oils before and after the thermo-degradation processes, and fried French fries was analyzed. The addition of HLRME affected the oxidation stability of refined rapeseed oil heated for 8 h daily for 3 days at 180 °C by preventing an increase in the peroxide values (PV), anisidine values (*p*-AnV), TOTOX and INTOX indexes, conjugated dienes (K_232_), and total polar material (TPM). However, thermal degradation generated similar amounts of conjugated trienes (K_268_) in non-supplemented and supplemented rapeseed oils. Fortified rapeseed oils after each heating cycle and French fries fried in them revealed higher antioxidant properties than those prepared in refined rapeseed oils without HLRME. Results from the present study suggest that HLRME, as a potential source of natural antioxidants from oil industry by-products, can prevent the degradation of refined rapeseed oil and help improve the quality of French fries.

## 1. Introduction

In Poland, rapeseed oil is the predominant edible oil extensively utilized in households, restaurants, and the food industry. Rapeseed oil is recovered from rapeseed, which belongs to the Brassicaceae family. It is abundant in unsaturated fatty acids, including linoleic acid (omega-6) and α-linolenic acid (omega-3), in a unique ratio of 2 to 1 and low levels of saturated fatty acids, resulting in a plethora of health benefits [1]. It also contains a high content of endogenous active components, such as tocopherols, phenolic compounds, and phytosterols [2]. Although polyunsaturated fatty acids (PUFA, C18:2 = 15–30% and C18:3 = 5–14%) present in rapeseed oils can be prone to oxidation reactions during thermo-degradation processes, the high amounts of natural antioxidants and the addition of natural plant extracts into these frying oils were frequently proposed by many researcher groups to exert positive effects by avoiding chemical alterations during heating [3,4,5,6,7,8,9,10]. Lately, to select an appropriate frying oil, consideration is given not only to sociocultural and economic factors but also the nutritional (oils with low amounts of saturated fatty acids—SFA and high amounts of monounsaturated fatty acids—MUFA) and technological (solidification possibility of oil with high SFA and pipe blockage in industrial frying) points of view [4]. Therefore, rapeseed oil is selected as commonly used for frying worldwide due to its stability, often enhanced by naturally present and added antioxidants, and its ability to preserve the color, original flavor, and nutritional profile. However, the optimum frying conditions and oil stabilization by using natural antioxidants should be chosen to limit the generation of degradation products, which can be absorbed by fried foods and directly affect their safety, quality, and consumers’ health.

Deep-frying and pan-frying processes are widely used in home cooking for food preparation because they impart desirable crispness, palatable and enticing taste, and texture [11,12]. This cheap and fast culinary technique involves a complex interplay of heat and mass transfer between the frying medium, generally vegetable oils and fried food products, changing their sensory and nutritional characteristics. However, all changes in frying oils and fried foods (physical: food dehydration, food lipid solubilization, and frying oil uptake and chemical: thermo-oxidation, polymerization, hydrolysis, and isomerization) depend on many factors, such as the characteristics of oil and food types, surface/volume ratio of the oil, rate of air incorporation into the oil, temperature of a heating process, length of immersion, and the kind of frying container material [13].

Apart from desirable changes in fried foods, including the thermal destruction of microorganisms and enzymes and the reduction of water activity on the surface of the foods, transport of health-beneficial components (unsaturated fatty acids, vitamins, and polyphenols), starch gelatinization inside, protein denaturation and participation in Maillard-type reactions with reducing sugars or lipid oxidation carbonyls, enhancing flavor and color, the undesirable changes, among them absorption of the potentially toxic compounds (volatile chain—scission products, non-volatile oxidized derivatives, dimeric, polymeric, and cyclic substances), also Maillard reaction products, reduction and oxidation of food lipids and lipid-soluble vitamins in fried foods, were observed by many authors [3,4,11].

It is well known that the composition of fatty acids and the presence of active components affect the oxidation resistance of oils utilized in frying processes. Therefore, in recent years, there has been increasing interest in the supplementation of frying media with extracts rich in natural antioxidants like phenolic acids, diterpenes, flavonoids, catechins, anthocyanins, and tannins to preserve the frying oils from oxidative deterioration and prolong and improve their quality. These changes and implementations can help increase public demand for the nutritious foods that are part of healthy and sustainable diets. In this case, commonly used vegetable oils, including rapeseed, sunflower, soybean, palm, and olive oils before frying processes, were enriched with essential oils and natural phenolic antioxidants extracted from seeds of rowanberry (*Sorbus aucuparia*), crabapple (*Malus baccata*), *Lepidium sativum*, and *Aframomum corrorima*, wild rose hip with seed (*Rosae pseudofructus cum fructibus*), longan (*Dimocarpus longan*), orange, lemon, mandarin peels, olive tree (*Olea europaea*) leaves, olive fruits, mixture of pomegranate, orange, and beetroot leaves, rosemary leaves, apple pomace, olive mill wastewater, *Camellia oleifera* seed cake, and blueberry, sunflower oil, and olive oil by-products [3,5,6,11,12,14,15,16,17,18,19,20,21,22,23,24,25,26,27].

Increasingly, attention has been paid to recovering antioxidant components from residues/by-products generated by agrifood industries. Among them, rapeseed meal is a by-product of rapeseed oil production rich in bioactive compounds, mainly sinapic acid derivatives, but also other phenolic acids (ferulic, coumaric, and p-hydroxybenzoic), flavonoids, and tannins. Notably, the polar nature of this oil industry residue versus the nonpolar character of oil indicates significantly higher amounts of polar bioactive components in rapeseed meal than in rapeseed oil [28,29].

However, phenolic compounds with antioxidant properties linked to cell wall constituents are non-extractable by organic solvents and remain in the solid rapeseed meal residue, leading to substantial waste. It is well known that these chemical bonds between bioactive compounds and cell wall components and the matrix of rapeseed meal can be broken after hydrolyses with acid, alkaline, or enzymatic solutions, which is beneficial for the valorization and commercialization of the obtained rapeseed meal hydrolysates rich in released phenolic compounds identified by chromatographic methods. Some research groups have utilized chemical hydrolysis using bases or acids as a comparatively economical, easy-to-control process to recover bound phenolic compounds from rapeseed meal [28,30,31,32,33].

Recently, rapeseed meal has been utilized to produce high-value products such as lipophilic antioxidants (sinapate alkyl esters), proteins, and monosugars simultaneously [34].

Expectedly, the dispersibility and solubility of polyphenols in the oil matrix determine their antioxidant properties. The antioxidant effects of protecting frying oil from thermal oxidation are the effective contact between the phenolic compounds and the unsaturated triacylglycerols (TAGs), which are assisted by molecular thermal motion.

For this reason, in our previous studies, the esterification of hydroxycinnamic acids with various alkyl alcohols has been used to enhance the hydrophobicity of phenolic acids and their antioxidant properties [35]. The obtained lipophilized phenolic compounds (phenolipids: octyl sinapate, octyl ferulate, octyl caffeate, cetyl sinapate, and cetyl ferulate) as new functionalized antioxidants had a beneficial effect on the antioxidant properties of fried French fries and absorbed oil, as well as oil uptake during frying [36]. Amphiphilic phenolipids added to rapeseed oil had appropriate hydrophobicity for concentrating at the oil–water interfaces where oxidation occurs during the fries frying process to exert their antioxidant effect effectively. Moreover, the canolol-enriched extracts extracted from the rapeseed meal improved the thermal stability, quality, and sensory attributes of the fortified rapeseed oils after multiple frying cycles [7,8].

However, to the best of our knowledge, there are no studies of the utilization of acid and alkaline hydrolysis of whole rapeseed meal to produce phenolic hydrolysates, which can be further valorized as natural antioxidants to enhance the physicochemical parameters of supplemented rapeseed oil and the antioxidant potential of fried fries.

Therefore, this work proposes the valorization of the productive chain from the investigation of total antioxidant compounds in hydrolyzed extracts, allowing the antioxidative description of a rapeseed by-product that was directed to the fortification of the final product, such as rapeseed oil utilized as a frying medium. In the first step of this study, the antioxidant properties of hydrolysates obtained from rapeseed meal, which is an oil-industrial by-product after acid and alkaline hydrolysis and lyophilization, were estimated. In the second step, the impact of acid-hydrolyzed and lyophilized rapeseed meal extract (HLRME) on oxidative stability, antioxidant activity (AA), and total phenolic content (TPC) in the refined rapeseed oil before and after pan-frying French fries was investigated. Finally, the AA and TPC in French fries fried in enriched rapeseed oil were determined by 2,2′-azino-bis(3-ethylbenzothiazoline-6-sulfonic acid) (ABTS), 2,2-diphenyl-1-picrylhydrazyl (DPPH), ferric reducing antioxidant power (FRAP), and Folin–Ciocalteu (FC) methods, respectively. Moreover, principal component analysis (PCA) and hierarchical cluster analysis (HCA), as unsupervised classification methods, and a correlation matrix were applied to assess the association between oxidative parameters and functional antioxidant properties of oils utilized for the frying process and fried French fries.

## 2. Materials and Methods

### 2.1. Reagents

All chemicals used in these studies were analytical or HPLC grade. 6-Hydroxy-2,5,7,8-tetramethylchromane-2-carboxylic acid (Trolox, TE, 97%), 2,2-diphenyl-1-picrylhydrazyl (DPPH, 95%), 2,2′-azino-bis(3-ethylbenzothiazoline-6-sulfonic acid) diammonium salt (ABTS), sinapic acid (SA, 98%), Folin–Ciocalteu (FC) reagent, and 2,4,6-tri(2-pyridyl)-s-triazine (TPTZ, 99%) were purchased from Merck (Warszawa, Poland). Hydrochloric acid (HCl), acetic acid (CH_3_COOH), sodium hydroxide (NaOH), iron(III) chloride hexahydrate, sodium carbonate, chloroform, petroleum ether (40/60), ethanol, methanol, and *n*-hexane were supplied from Alchem (Toruń, Poland). All solutions were prepared with redistilled water.

### 2.2. Materials

Rapeseed meal and refined rapeseed oil in the original packages (polyethylene bag and 1-L polyethylene terephthalate (PET) bottle, respectively) were kindly donated by the local vegetable factory. The frozen French fries (crinkle cut) pre-fried in sunflower oil (Aviko brand, purchased from a local supermarket) were selected as the standard test food for frying. Before experiments, rapeseed meal was kept in a cold and dark place, while oil was stored in a refrigerator at 4 °C.

### 2.3. Preparation of Hydrolyzed and Lyophilized Rapeseed Meal Extracts

**Figure 1:**
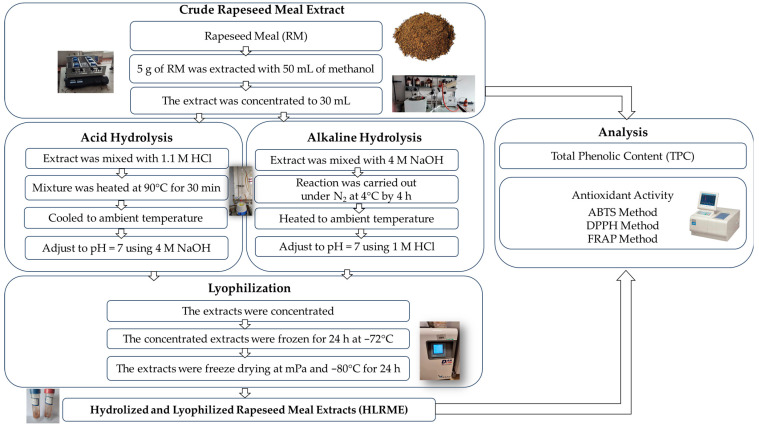
Flow chart of the preparation of hydrolyzed and lyophilized rapeseed meal extracts and their analysis.

A flow chart showing the overall preparation of hydrolyzed and lyophilized rapeseed meal extracts and their analysis is shown in Figure 1.

#### 2.3.1. Crude Rapeseed Meal Extract

The extraction of phenolic compounds from rapeseed meal was carried out using the method described by Siger et al. [30] with some modifications. In brief, 5 g of ground rapeseed meal was mechanically extracted with 10 mL of methanol for 30 min, using an SHKA 2508-1CE shaker (Labo Plus, Warszawa, Poland). Then, the sample was centrifuged at 5800 rpm (centrifuge MPW-54, MPW MED. INSTRUMENTS, Warszawa, Poland) for 10 min and transferred quantitatively into a round bottom glass. The extractions were repeated thrice, and the combined extracts were concentrated up to 30 mL by rotary evaporation.

#### 2.3.2. Alkaline Hydrolysis and Acid Hydrolysis of Rapeseed Meal Extract

Sodium hydroxide was used for the alkaline hydrolysis of rapeseed meal extract, while acid hydrolysis of this extract was carried out with hydrochloric acid according to procedures described by Siger et al. [30] with slight modifications. The methanolic extract of rapeseed meal was divided into two parts, 15 mL each, to prepare alkali and acid hydrolysates. In the case of alkaline hydrolysis, 18 mL of 4 M NaOH was added to the methanolic extract in the round bottom flask. Hydrolysis was carried out under nitrogen with stirring at 4 °C for 4 h. Then, the reaction mixture was adjusted to pH = 7 using 1 M HCl. The second part of the methanolic rapeseed meal extract was transferred into a round bottom flask and mixed with 18 mL of 1.1 M HCl for acid hydrolysis. The solution was flushed with nitrogen, stirred, and heated at 90 °C for 30 min. After cooling, the mixture was adjusted to pH = 7 using 4 M NaOH.

#### 2.3.3. Lyophilization of Alkaline- and Acid-Hydrolyzed Rapeseed Meal Extracts

At first, alkaline- and acid-hydrolyzed rapeseed meal extracts were concentrated under reduced pressure at 55 °C using a rotary evaporator (Unipan 350P; Warszawa, Poland). Then, the obtained extracts were frozen for 24 h at −72 °C and were dried using the freeze-drying machine LyoQuest (Telstar, DAN LAB, Białystok, Poland) at mPa and −80 °C for 24 h.

### 2.4. Preparation of Rapeseed Oil Enriched with Acid-Hydrolyzed Rapeseed Meal Extract

The acid-hydrolyzed and lyophilized rapeseed meal extract (HLRME) was added to refined rapeseed oil and placed in an ultrasonic cleaner bath (Sono Swiss, SW 6H, Labo Plus, Warszawa, Poland) with ultrasound input power of 180 kW for 15 min to obtain a final HLRM concentration of 200 ppm. Test refined rapeseed oil without any antioxidant addition was considered as control.

**Figure 2:**
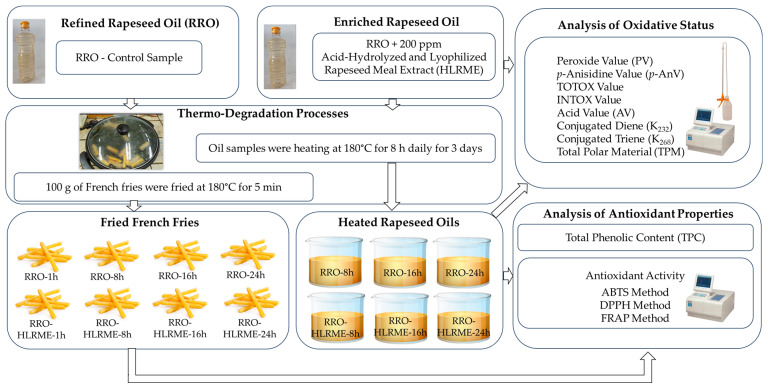
Flow chart of enrichment rapeseed oil preparation, heat treatment, and frying French fries.

A flow chart of enrichment rapeseed oil preparation, heat treatment, and frying French fries is depicted in Figure 2.

### 2.5. Frying Test

The refined rapeseed oil enriched with HLRME and non-supplemented refined rapeseed oil were utilized to fry frozen French fries (approximately 9 × 9 × 30 mm). These experiments were carried out using a commercial round electric frying pan (diameter = 32 cm) with a non-stick TEFLON^®^ (Wilmington, DE, USA) coating and a glass lid. The electric pan had a total electrical power of 1500 W and the possibility to regulate it at 5 heating levels (CLATRONIC PP 3401, Kempen, Germany). The oil temperature was continuously monitored using a thermometer. At first, about 550 g of each oil (1.5 cm oil height) was placed into the pan and preheated up to 180 °C. In these conditions, the refined rapeseed oil supplemented with HLRME and control oil without HLRME were heated for 8 h daily for 3 days. The experiment was stopped after 8 h and restarted the next day without an additional part of the oil. On the first day, after 1st and 8th hour of heating, two parts of 100 g of frozen French fries were fried in control and enriched oils for the optimal frying time of 5 min until a golden, crispy coating was obtained, while one portion of fries (100 g) was fried after 16 h and 24 h of heating each oil on the next two days. After frying, the fries were transferred to a sieve to drain any remaining oil adhered to the fries. Also, after each day, 40 g of oil samples was collected in amber-colored capped bottles at the end of each heating day and frozen until further analysis. After 3 days of the experiment, the remaining oil layer height was around 1 cm. Initially, the ratio of French fries to the oil was about 0.18, while in the end heating process, this ratio increased to approximately 0.27.

### 2.6. Determination of Oxidative Status of Oil Samples

The oxidative status of supplemented and non-supplemented refined rapeseed oils before and after frying processes was characterized using the official methods to analyze peroxide value (PV), *p*-anisidine value (*p*-AnV), acid value (AV), and conjugated polyenes.

The PV was determined iodometrically (visual titrimetric procedure) and expressed as milliequivalents of active oxygen per kilogram of oil (meq O_2_/kg) according to the standard procedure PN-EN ISO 3960:2017-03 [37]. The *p*-AnV was characterized spectrophotometrically based on the official ISO 6885:2016 method [38]. These two parameters, PV and *p*-AnV, were combined to estimate the total oxidation of TAGs and given as TOTOX = (2 × PV) + *p*-AnV. The integral oxidation (INTOX value), calculated as follows: INTOX = (2 × *p*-AnV) + PV, was also used as an alternative to the TOTOX value. This indicator focuses on the loss of peroxides through their decomposition into secondary oxidation products in the advanced stages of oxidation [39].

However, AV was assessed to measure the degree of oil hydrolysis using the ISO 660:2020 method [40].

Moreover, the specific extinction coefficients (K_232_ and K_268_) indicating amounts of conjugated dienes (CD) and conjugated trienes (CT), respectively, were evaluated spectrophotometrically as the absorbance of 1% oil solution in *n*-hexane at 232 and 268 nm, according to the ISO 3656:2011 method [41].

### 2.7. Determination of Total Polar Material in Oil Samples

The sample of each studied oil was collected every 8 h, and their total polar material (TPM) was measured directly after sampling using a Testo™ 270 cooking oil tester (Testo, Lenzkirch, Germany) according to the manufacturer’s instructions.

### 2.8. Determination of Antioxidant Activity and Total Phenolic Content

#### 2.8.1. Preparation of Samples

Before the determination of the AA and TPC in the crude and alkaline- and acid-hydrolyzed and lyophilized rapeseed meal extracts, their fresh solutions (c = 0.5–1.25 g/L) were obtained in methanol. Moreover, the methanolic extracts of rapeseed oils with and without HLRME before and after the frying processes and fried French fries were prepared according to the procedure described in our previous work [36]. Briefly, 3.00 g of each oil sample and 2.00 g of fried French fries previously ground in a laboratory grinder were weighed on an analytical balance and transferred into test tubes and 50 mL Erlenmeyer flasks, respectively. Then, these samples were extracted with 5 mL and 10 mL of methanol, respectively, for 30 min at ambient temperature using an orbital shaker (SHKA25081 CE, Labo Plus, Warszawa, Poland). The oil mixtures were frozen (−20 °C, 30 min), and the French fry mixtures were centrifuged for 15 min (centrifuge MPW-54, MPW MED. INSTRUMENTS, Warszawa, Poland) to separate the obtained methanolic extracts from samples. The extraction procedure was repeated three times for the same studied sample with fresh portions of methanol. The resultant extracts were collected and stored in glass bottles in a refrigerator until the AA and TPC measurements were carried out.

#### 2.8.2. Procedures of Analytical Methods

The scavenging activity against DPPH and ABTS free radicals and the capacity of antioxidants present in investigated samples to reduce ferric to ferrous ions by an electron transfer process (FRAP method) were measured according to procedures described in our previous article [42] with slight modifications. Moreover, the Folin–Ciocalteu (FC) colorimetric method, reported in detail in the same work, was utilized for the analysis of TPC in the prepared methanolic extracts.

The AA and TPC spectrophotometric measurements were conducted using a Hitachi U-2900 spectrophotometer (Hitachi, Tokyo, Japan) in a 1 cm quartz cell. The AA and TPC results were expressed as micromoles of Trolox equivalent and sinapic acid equivalent, respectively, per 1 g of crude and hydrolyzed rapeseed meal extracts (µmol TE/g and mg SAE/g) and 100 g of oil and fries samples (µmol TE/100 g and mg SAE/100 g).

### 2.9. Statistical Analysis

All analyses were carried out in triplicate. The results were presented as mean (c) ± standard deviation (SD). One-way analysis of variance (ANOVA), followed by the Duncan test, was performed to analyze the significant differences between data (*p* < 0.05).

Moreover, data were subjected to chemometric analyses as principal component analysis (PCA), hierarchical cluster analysis (HCA) with Ward’s method using Euclidean distances, and color matrix of correlations to explore similarities and differences between analyzed oils and fried French fries or project these objects in a two-dimensional factor plane based on various oxidative and antioxidant characteristics.

Statistical analysis of data was performed using the IBM SPSS Statistics 29 software (IBM, New York, NY, USA) and Statistica 8.0 software (StatSoft Inc., Tulsa, OK, USA).

## 3. Results and Discussion

### 3.1. Effect of Chemical Hydrolysis on Antioxidant Properties of Rapeseed Meal Extract

Two radical scavenging activity methods, ABTS and DPPH, and one FRAP method, based on the reduction power, were proposed to evaluate the AA of the prepared rapeseed meal extracts because different analytical tests involving other mechanisms better characterize antioxidant features [43]. Moreover, the TPC in these extracts before and after acid hydrolysis and alkaline hydrolysis was analyzed utilizing an FC assay. The obtained TPC and AA results for the studied rapeseed meal extracts are listed in Table 1.

Notably, the crude extract contained the lowest TPC (12.20 mg SAE/g) due to the presence of complex phenolic components, which cannot be detected and measured by the FC spectrophotometric method [44]. A chemical hydrolysis pretreatment method caused the decomposition of complex phenolic compounds to the simpler components and significantly increased TPC by about 1.5-fold for alkaline hydrolysate and 2-fold for acid hydrolysate compared to the crude rapeseed meal extract (Duncan test, Table 1). The same tendency for the amounts of total phenolics in crude extract of *Brassica napus* L. seed meal (1577–1705 mg SAE/100 g), after alkaline hydrolysis (1608–1760 mg SAE/100 g) and acid hydrolysis (1689–1807 mg SAE/100 g), was observed by Siger et al. [30]. These authors indicated that chemical hydrolysis released free phenolic acids (mainly sinapic acid isomers) with higher antioxidant potential than their related forms, including esters or glucosides.

Furthermore, the Duncan test indicated that the type of hydrolysis caused significant differences in the AA of the obtained extracts analyzed by ABTS, DPPH, and FRAP assays (Table 1). Notably, the ABTS values of the studied rapeseed meal extracts after alkaline and acid hydrolysis were 5.8-fold and 4.6-fold higher than that of the ABTS of crude extract. This suggests that rapeseed meal extract contained higher amounts of ester and ether bonds between the phenolic compounds and the cell walls breaking down during alkaline hydrolysis than glycosidic bonds hydrolyzing during acid hydrolysis [45]. Surprisingly, insignificant differences in DPPH results were observed for rapeseed meal extract before and after acid hydrolysis (Duncan test, Table 1). In contrast, extract after alkaline hydrolysis had approximately eight times higher DPPH than crude extract. This confirms that alkaline hydrolysis allowed the breaking of the bonds of esters and the release of the simple phenolic compounds from the cell walls, which were able to scavenge DPPH radicals.

On the contrary, a higher FRAP value for rapeseed meal extract after acid hydrolysis (650.24 μmol TE/g) than after alkaline hydrolysis (586.47 μmol TE/g) provided information on glycosylated phenolics in rapeseed meal, which after releasing more easily reduced Fe(III) to Fe(II) by donating an electron than those from disrupted esters and contributed more significantly to the FRAP results.

Unexpectedly, the alkaline hydrolysis of canola meal extract using sodium hydroxide caused a decrease in TPC values from 11,183 µg SAE/g down to 8052 and 8678 µg SAE/g for non-hydrolyzed 80% methanolic extract and after 2 M and 4 M NaOH treatment, respectively, probably due to the decomposition of vinylsyringol and sinapic acid [32]. Nevertheless, our previous work concerning the effect of acid and alkaline hydrolysis on the antioxidant properties of black cumin meal demonstrated higher AA and TPC in the meal extract after acid hydrolysis (DPPH = 35,629 μmol TE/100 g, CUPRAC = 12,601 μmol TE/100 g, TPC = 691 mg gallic acid equivalent (GAE)/100 g) than after alkaline hydrolysis (DPPH = 2539 μmol TE/100 g, CUPRAC = 5959 μmol TE/100 g, TPC = 613 mg GAE/100 g) [45].

Moreover, Pag et al. [46] noticed that the TPC and radical scavenging ability of 60 and 80% crude ethanolic extracts from flaxseed cake measured by the DPPH test increased upon acid hydrolysis (TPC = 617–1115 mg GAE/L and DPPH = 41–73%) probably due to disruption of linkages and deglycosylation of the phenolic compounds.

Many researchers concluded that acid and alkaline hydrolysis can liberate bioactive compounds from the food matrix into the liquid fraction, which often present as insoluble-bound complexes coupled with cell wall polymers. This phenomenon provides an increase in free phenolic compounds depending on the type and proportion of covalent bonds between polyphenol compounds and cell wall polymers [47].

The reason for the choice of acid-hydrolyzed and lyophilized rapeseed meal extract (HLRME) to enrich refined rapeseed oil used as a frying medium was the highest TPC (24.80 mg SAE/g) and FRAP (650.24 μmol TE/g) among the prepared rapeseed meal extracts, which is attributed to the presence of hydrophilic phenolic compounds, such as sinapic acid derivatives. It is well known that the various oil–water interfaces formed by trace amounts of moisture in bulk oil during frying of frozen products and air–oil interfaces are important sites for lipid oxidation. However, hydrophilic antioxidants protect more effectively against lipid oxidation than lipophilic antioxidants because the latter are dissolved in the oil, while polar antioxidants can be distributed and remain located at these interfaces [14].

### 3.2. Changes in Antioxidant Properties of Refined Rapeseed Oils Without and with Hydrolyzed and Lyophilized Rapeseed Meal Extract During Heating

The decreasing trends in TPC, ABTS, DPPH, and FRAP results for refined rapeseed oils before and after the addition of HLRME with the prolonged heating time and frying of four portions of French fries are presented in Table 2.

This can be explained by the thermal degradation of antioxidants present in non-supplemented and supplemented rapeseed oils. However, adding HLRME rich in antioxidants reduced the oxidative processes in oils due to their potential to act by inhibiting thermal oxidation.

Antioxidants are crucial factors in preventing the oxidation process by neutralizing radical species. The frying processes hugely impact the level of antioxidant compounds, including phenolic acids, flavonoids, tocopherols, and vitamins, both in the frying media and the products. The amounts of antioxidants in chosen types of oils and foods for frying are closely related to these naturally present and generated during heating processes [48].

Our results confirmed that the TPC and AA results for fortified rapeseed oils determined by ABTS, DPPH, and FRAP were significantly higher than those for pure refined rapeseed oils (Duncan test, Table 2). However, each heating cycle decreased the TPC and AA of oils without and with HLRME. The 24 h of thermal degradation and frying of four portions of French fries reduced TPC by 67% and 68% for non-supplemented and supplemented oils, respectively, whereas the AA values of rapeseed oils without and with HLRME were lower by about 75–91% and 69–86%, respectively, after this heating time. The TPC results indicate an initial increase in the phenolic compounds in rapeseed oil after HLRME supplementation without limiting their degradation during frying processes. On the other hand, the thermal-sensitive antioxidants naturally present in oils and incorporated with HLRME, determined as AA by the proposed analytical methods, were probably reduced. However, the inhibition effect of antioxidant destruction in oils was observed after the enrichment of HLRME.

The results of the first experimental phase (8 h heating) demonstrated the highest decrease in TPC and AA (about 2.0–3.5 times) both for non-supplemented and supplemented oils. The extension of heating time from 16 to 24 h did not demonstrate such a drastic loss of antioxidants from the frying media, except for antioxidants determined by the FRAP method. This fact confirms that the hydrophilic antioxidants capable of reducing colorless Fe(III)-TPTZ into intense blue Fe(II)-TPTZ complex were easily thermally degraded compared to hydrophobic antioxidants, which can be analyzed by ABTS and DPPH methods. Therefore, selecting the appropriate analytical method is crucial to accurately understand the antioxidant effect on oil during frying, which is based on the complex lipid oxidation mechanisms.

It is necessary to emphasize that oxidation and thermal reactions occur simultaneously due to the effect of temperature during frying processes. Nevertheless, the thermal oxidation mechanism is the same as the chemical autoxidation and consists of three steps: initiation, propagation, and termination. However, the oxidation processes during food frying are more complex than those under oil heating due to introducing food products into oil and their chemical changes, including dehydration, protein denaturation, and starch gelatinization. During the frying process of edible oil, lipid oxidation involves chain reactions of free radicals, whose amounts depend on oil fatty acid composition and frying time [49]. Therefore, phenolic compounds can inhibit the oxidation process during frying according to three possible mechanisms: (1) scavenging free radicals, (2) scavenging carbonyl to trap harmful aldehydes formed during frying, and (3) a non-radical mechanism that the proposed acid-catalyzed polymerization and dimerization could explain the protective action of phenolic compounds [50].

Most recent research has focused on the application of exogenous phenolic compounds due to the insufficient amount of endogenous antioxidants to delay the decrease in oil quality under frying conditions. Among them, phenolic compounds with hydroxyl groups remaining in by-products of the oil industry after oil extraction, such as oil cakes and meals, were used to improve the thermal stability of vegetable oils during frying [50]. Interestingly, increasing temperature increased the protective effect of phenolic compounds due to their higher solubility or dispersal under heating or frying processes.

For comparison, tea polyphenol extract (0.04%) had an inhibitory impact on the deterioration of rapeseed oil quality during frying of potato slices after 24 h at 180 °C [9]. These authors observed a more significant increase in TPC after the fortification of rapeseed oil with tea polyphenols (from 108.51 to 518.91 mg GAE/kg) when compared to our TPC results (Table 2). Expectedly, TPC in oil samples significantly decreased with the increase in frying time within the first 8 h, while the TPC in fortified oil was higher than that in the control sample without tea polyphenols, similar to our observation. This indicates that tea polyphenols protected the oil quality during frying processes because they exhibited antioxidant activity, which can effectively block the free radical reaction by transferring the hydrogen proton to radicals at the initiation and/or quenching some radicals at the propagation step.

Moreover, the efficacy of extracts from olive leaf, hazelnut leaf, and hazelnut green leafy cover on TPC and AA of canola oils fried for seven consecutive days was evaluated [51]. These authors found about a 5- to 10-fold increase in AA (529.51–1214.20 μmol TE/g) after adding prepared extracts compared with the control sample (112.43 μmol TE/g). The AA values of non-supplemented and supplemented (200 ppm) canola oils were decreased by frying days, but even at the end of 7 days, the AA of the enriched oil samples (109.90–199.80 μmol TE/g) was higher than in the first days of a control sample (103.90 μmol TE/g). Unexpectedly, phenolics were not detectable in canola oil at the beginning of frying, whereas TPC ranged between 0.02–0.08 mg GAE/g and 0.01–0.06 mg GAE/g for oils fortified with olive leaf and hazelnut green leafy cover until the end of 7 days frying. However, all phenolics measured by the FC method were lost in oils with hazelnut leaf extract after the third frying day.

### 3.3. Changes in Oxidative Status of Refined Rapeseed Oils Without and with Hydrolyzed and Lyophilized Rapeseed Meal Extract During Heating

The changes in oxidative status indicators (PV, *p*-AnV, TOTOX, INTOX, AV, K_232_, K_268_, and TPM) of refined rapeseed oils without and with HLRME during the whole thermal degradation and frying processes of French fries are presented in Table 3. The Duncan test indicated that all analyzed oxidative parameters of the investigated oils significantly increased with prolonged heating time.

#### 3.3.1. Peroxide Value

It is well known that peroxide value is used to estimate the primary oxidation products, such as peroxides and hydroperoxides, in frying oil. Therefore, fresh oils without and with HLRME before the thermo-degradation process had the lowest PV values (0.18 and 0.36 meq O_2_/kg), which are below the legal limit (PV < 5 meq O_2_/kg) permitted for vegetable refined oils. It can be noted that the differences between PV results for oils non-supplemented and supplemented with HLRME were insignificant before starting the heating experiments (Duncan test, Table 3). However, somewhat higher amounts of hydroperoxides in fortified rapeseed oil can be explained by the formation of primary oxidation products during intensive mixing of oil with HLRME. Nevertheless, after the first cycle (8 h) of heating and French fries frying, the amounts of hydroperoxides in oils (PV = 14.15 meq O_2_/kg and PV = 8.73 meq O_2_/kg for control and fortified rapeseed oil, respectively) exceeded the permissible value. The further prolonged heating time of up to 24 h demonstrated a continuous rise of PV indicators to 17.16 and 16.40 meq O_2_/kg for oil samples without and with HLRME, respectively. Evidently, the addition of HLRME to refined rapeseed oil caused a slower increase in the levels of primary oxidation products, proving the higher thermo-oxidative stability of fortified oil. At the end of the thermo-degradation process, rapeseed oil enriched with HLRME had a significantly lower PV than the control oil (Duncan test, Table 3). Therefore, the antioxidants present in HLRME are crucial to interpreting their efficiency in inhibiting the formation of primary oxidation products. Despite the 24 h heating process and the susceptibility of the peroxides and hydroperoxides to decomposing due to their unstable nature, leading to the generation of low molecular secondary oxidation compounds such as aldehydes, ketones, alcohols, and free fatty acid, a decrease in PV indicators in the last step of these experiments was not recorded. The peroxides in these conditions probably still formed faster than they degraded.

For comparison, canolol extracted from canola meal and phenolic extracts from canola oil deodistillates were proposed as efficient antioxidant ingredients for canola oil protection against the formation of peroxides during the frying of French fries for 5 days (6 h per day) [10]. The added canolol reduced the formation of primary oxidation products in canola oils more effectively than phenolic extracts. On the 5th day of frying, the PV results were 110.30, 207.91, and 120.00 μM, respectively, for canola oils enriched with canolol extract, phenolics extract from oil deodistillates, and control canola oil sample without any added antioxidants. In contrast to our study, these authors observed a decrease in hydroperoxide contents in canola oils with prolonged frying time, indicating their decomposition into secondary oxidation products.

Moreover, the antioxidant efficacy of ethanolic olive cake extract added to sunflower oil at levels of 100, 200, 400 and 600 ppm heated at 180 °C for 4 h/day for 5 consecutive days on generation of primary lipid oxidation products was investigated by Abd-ElGhany et al. [52]. The PV indicators for each oil sample increased with the heating time, whereas sunflower oil without olive cake extract had the highest PV (1.98–7.12 meq O_2_/kg) at each heating time and the end of heating. The fortification of sunflower oil with increasing concentrations of olive cake extract caused a successive decrease in PV results (1.35–6.85, 1.20–6.30, 1.10–6.05, and 1.01–5.88 meq O_2_/kg, respectively, for oils with 100, 200, 400, and 600 ppm of extract).

In other studies, prepared extracts from various spices, herbs, plant leaves, seeds, fruits, agriculture and processing by-products, and synthesized phenolipids also effectively inhibited the thermo-oxidative primary oxidation reactions of vegetable oils, including rapeseed oil, under frying conditions [36,49,53].

#### 3.3.2. Anisidine Value

The primary oxidation products, peroxides, and hydroperoxides are transferred into secondary oxidation products, in which aldehydic components like 2,4-dienals and 2-alkenals are determined as the *p*-anisidine value (*p*-AnV). Sometimes, *p*-AnV results were associated with the sensory scores of oils because carbonyl compounds measured as *p*-AnV were responsible for the rancid flavor of frying oil [7,10].

It is noteworthy that the amounts of secondary oxidation products in refined rapeseed oils without (*p*-AV = 1.37) and with HLRME (*p*-AnV = 1.08) before frying French fries did not exceed the legal limit (*p*-AnV < 8). However, *p*-AnV results rapidly increased after the first heating cycle, reaching 104.88 and 111.41 for enriched and control oils, respectively. As presented in Table 3, significant increases (Duncan test) in *p*-AnV were observed in both oils with each successive heating, but the control oil samples had higher *p*-AnV values at each heating period. This suggests that adding antioxidants present in HLRME remarkably delayed the formation of secondary oxidation products of the supplemented oils with prolonged thermal degradation.

A similar protective effect of added antioxidants from other oilseed by-products on generating secondary oxidation products in enriched oils during frying was reported. Wu et al. [26] found that the added *Camellia oleifera* seed cake extract significantly reduced *p*-AnV (50–200) of soybean oils heated from 220 to 840 min compared with the control soybean oil (*p*-AnV = 90–230). For this reason, after frying potato strips for 840 min at 180 °C, amounts of oxidative secondary degradation products in soybean oil with the *Camellia oleifera* seed cake extract were lower than those in control soybean oil, indicating that the extract introduced into the oil had good stability and high oxidative inhibition effect.

Moreover, higher concentrations of canolol-enriched extract (200, 500, and 750 mg/kg) obtained from the extraction of fluidized bed-treated canola meal were more effective in preventing the formation of secondary oxidation products in high-oleic canola oils during deep-fat frying for 30 h [7]. At the end of frying, the control oil reached a *p*-AnV of above 100, while the *p*-AnV results for the enriched canola oils ranged between 25 and 46 depending on the added concentration of canolol-enriched extract. Similarly, on the 5th day of frying, the *p*-AnV (221.72 mmol/kg) of refined canola oil was significantly higher than the *p*-AnV results (98.83 and 79.90 mmol/kg) for oils incorporated with phenolic extracts from canola oil deodistillates and crude canolol extracted from canola meal, respectively [10].

All *p*-AnV results indicate that supplementation of edible oils with phenolic compounds extracted from by-products of the oil industries could substantially improve the quality of frying oils by inhibiting the production of secondary oxidation products during the frying processes.

#### 3.3.3. Total Oxidation Index and Integral Oxidation Index

The TOTOX values of non-supplemented and supplemented rapeseed oils were evaluated because they are good indicators of their overall oxidation occurring during frying processes. However, the TOTOX value cannot effectively represent the oxidation of advanced oxidative processes such as thermo-degradation and frying. Therefore, a new indicator referred to as the Integral Oxidation Value (INTOX index), which is more effective than the TOTOX value for assessing thermo-degradation states of refined rapeseed oils without and with HLRME during 24 h heating, was calculated (Table 3). The INTOX indicator prioritizes the formation of secondary oxidation compounds when primary oxidation products decrease due to a lower formation rate and higher degradation rate in advanced oxidation processes [39].

It can be noted that the TOTOX indexes of fresh, refined rapeseed oils without and with HLRME were calculated to be 1.73 and 1.80, respectively (Table 3). They thus did not exceed the TOTOX value (<20) recommended by the German Guidelines for Edible Fats and Oils, suggesting the high quality of oils before frying processes [54]. The studied oils revealed a similar trend in the increasing TOTOX indexes as PV and *p*-AnV values with increased heating times. The first heating cycle (after the 8th hour) caused the highest enhancement of TOTOX, to 139.71 and 122.34 for non-supplemented and supplemented rapeseed oils, respectively. However, the TOTOX reached the maximum levels of 221.10 and 196.18 for control oil and oil fortified with HLRME after 24 h of heating and fourth fries portion frying.

In both the control oil samples and oils with HLRME, the INTOX values experienced a notable increase after the thermo-degradation processes, from 2.92 to 390.72 for non-supplemented oils with high *p*-AnV results (1.37–186.78) and from 2.52 to 343.16 for enriched rapeseed oils having lower amounts of secondary oxidation products (*p*-AnV = 1.08–163.38). It is noteworthy that the longest heating time of the studied oil samples caused the highest INTOX values, but enriched rapeseed oils had lower INTOX indexes compared to those obtained for control oil samples.

The TOTOX and INTOX results listed in Table 3 indicate that HLRME could effectively delay the primary and secondary oxidation degree of heating rapeseed oil and enhance its stability.

Another study has also reported the highest TOTOX values of 1778.4, 762.97, and 408.07 for control oil and oils loaded with phenolic extracts from canola oil deodistillates and canolol extracted from canola meal, respectively, after the first day of frying [10]. In addition, all rapeseed oils without and with five novel synthetic antioxidants: octyl sinapate, octyl ferulate, octyl caffeate, cetyl sinapate, and cetyl ferulate, tended to increase total oxidation after frying processes (TOTOX = 1.67 and 31.22 for control oil, TOTOX = 1.92–4.91 and 15.37–23.79 for oils with phenolipids before and after frying, respectively) [36]. Nevertheless, the presence of phenolipids in rapeseed oils used for frying French fries significantly reduced their total oxidation and improved quality.

For comparison, the addition of essential oil mixtures: oregano and laurel essential oils (0.05% and 0.05%), oregano and peperine essential oils (0.05% and 0.02%), and oregano essential oil at a concentration of 0.02% to sunflower oil, caused a decrease in INTOX indicators (430, 670, 660, respectively) compared with control oil sample (INTOX = 680) after exposure at 60 °C for 28 days [39].

Moreover, the values of the linear regressions used for the four oxidation parameters representing primary (PV) and secondary (*p*-AnV) oxidation products, total oxidation (TOTOX), and integral oxidation (INTOX) state of the tested oils are listed in Table 4.

It can be noted that higher slope values were calculated for the *p*-AnV (7.42), TOTOX (8.70), and INTOX (15.48) of refined rapeseed oils without HLRME than those of enriched oils (β_1_ = 6.42, 7.75, and 13.51 for *p*-AnV, TOTOX, and INTOX, respectively). In contrast, the slope of PV (0.64) for the control sample was insignificantly lower than that for the rapeseed oil supplemented with HLRME (β_1_ = 0.67). This suggests that supplemented oil exhibited a lower acceleration for the formation of secondary oxidation products than control rapeseed oil, while somewhat higher amounts of hydroperoxides were generated in oil with HLRME. However, the addition of HLRME caused an improvement in the fit of the linear regression model for PV, from 0.75 for control oil to 0.94 for fortified oil. Oppositely, *p*-AnV and INTOX values for supplemented rapeseed oil revealed an insignificantly lower fit of the regressions with values of 0.89 than R^2^ (0.92 and 0.91, for *p*-AnV and INTOX indicator, respectively) for control refined rapeseed oil. Meanwhile, the TOTOX index had an identical fit (R^2^ = 0.90) for both oil samples without and with HLRME. This confirms the HLRME addition did not significantly improve the overall oxidation state of rapeseed oil during thermo-degradation processes.

#### 3.3.4. Acid Value

Acid value (AV) is a crucial oil indicator used to measure oil deterioration due to the hydrolysis of TAGs. The increase in AV during the frying processes can be explained by the rise in free fatty acids (FFAs) formed by the hydrolysis of TAGs and the oxidative decomposition of unsaturated TAGs. Additionally, the enhancement of oil acidity can be caused by an increase in carboxyl-containing polymer formed by thermal or oxidative polymerization under heating conditions [4,13]. Importantly, as a weak nucleophile, water from frozen French fries during their frying can break the ester linkage of TAGs and produce di- and monoacylglycerols, glycerol, and free fatty acids, the amounts of which successively increase with the number of frying cycles [36,53]. Therefore, lower AV results after frying processes indicate higher stability and quality of the used frying media.

It is noteworthy that both tested oils without and with HLRME before and after frying revealed AV results ranging between 0.015 and 0.278 mg NaOH/g, which are below the recommended maximum level for refined oils (AV < 0.3 mg NaOH/g) [40]. It was observed that the AV of all oils increased as the heating duration was extended, while the AV increase rate was slowed down when HLRME was added. Moreover, an insignificant increment in the AV for supplemented rapeseed oil after the first heating period (8 h) was found (Duncan test, Table 3). The most prominent increase in the contents of free fatty acids was determined after the second heating cycle of utilized oils (AV = 0.036–0.141 mg NaOH/g and 0.026–0.103 mg NaOH/g for the control oil sample and oil fortified with HLRME, respectively). The further elongation of heating time caused a two-fold increase in AV in both oil samples. However, the obtained AV results demonstrated that adding HLRME with high antioxidant properties (Table 1) to rapeseed oil effectively delayed the release of FFAs from TAGs upon thermal degradation and frying fries (Table 3).

Similar trends of FFA enhancement in various vegetable oils (rapeseed oil, sunflower oil, soybean oil) enriched with phenolic extracts from tea, *Teucrium polium*, and oil industry by-products (canola meal, *Camellia oleifera* cake, olive waste cake) with increasing heating time were reported by other authors [7,9,26,52,53]. As in our study, at the first interval of frying, the FFA amounts in different frying oils rose slowly. However, continuous frying processes caused a significant increment in the AV results.

Gao et al. [9] demonstrated that tea polyphenols delayed oxidation and hydrolysis reactions in rapeseed oil heated for 0, 4, 8, 12, 16, 20, and 24 h, causing lower AV results (0.4–1.1 mg/g) for fortified oil than those (AV = 0.4–1.8 mg/g) for control oil. Moreover, canola oil without exogenous antioxidants heated for 30 h had a higher AV (0.12–1.05%) than oil containing 200–1000 ppm of *Teucrium polium* extract (AV = 0.07–0.50%) [53]. Contrary to our results, Wu et al. [26] showed that the addition of phenolic extracts from *Camellia oleifera* seed cake did not exhibit an inhibitory effect on the increase in FFA generation in soybean oil during frying up to 840 min. The same AV results varying from 0.2 to 1.2 mg/g were obtained for oils without and with *Camellia oleifera* seed cake extract. Similarly, there were insignificant differences between high-oleic canola oil without and with rosemary extract [7]. The AV for both oils increased from 0.1 to 0.6 g KOH/100 g. Surprisingly, the addition of canolol-enriched extracts to high-oleic canola oil in different concentrations (200, 500, and 750 mg/kg) resulted in a higher AV (0.36–1.0 g KOH/100 g) in the initial oils, depending on the extract concentration. The processes of deep-frying increased the accumulation of FFAs in oils fortified with canolol-enriched extracts, and after 30 h of frying, the AV results were 0.83, 1.63, and 2.20 g KOH/100 g for oils with 200, 500, and 750 mg of extract/kg. The authors explained this fact by the formation of FFAs during the preparation of canolol-enriched extracts in the presence of moisture in meal and extraction with supercritical carbon dioxide [7].

#### 3.3.5. Conjugated Dienes and Conjugated Trienes

The oxidation rate of heated oils can also be monitored by the changes in specific absorptivity at 232 and 268 nm, which measure the amounts of conjugated dienes (CD) and conjugated trienes (CT), respectively [10,55]. The K_232_ parameter is associated with forming primary lipid oxidation products, whereas the K_268_ parameter characterizes changes in secondary oxidation products such as carbonyl compounds (aldehydes and ketones).

Insignificant differences in CD concentrations (K_232_ = 0.562 and 0.498) were observed between fresh rapeseed oil samples without and with HLRME (Duncan test, Table 3). However, the first cycle of heating and pan-frying significantly increased the CD accumulation in both oils (K_232_ = 2.789 and 2.036 for control and enriched oils, respectively) due to the rearrangement of methylene-interrupted double bonds and generated more stable structures containing *trans* and conjugated double bonds after heat treatment. Interestingly, the K_232_ parameters of all oil samples insignificantly increased with longer heating cycles (Duncan test, Table 3). Moreover, the HLRME addition significantly inhibited the CD production in fortified oils during the thermo-degradation process (K_232_ = 2.036–2.144 and 2.770–2.814 for supplemented and non-supplemented oils, respectively).

Asadi and Farahmandfar [53] also observed an increase in CD levels in canola oils before and after the addition of *Teucrium polium* extract with increasing time of frying. The CD amounts (5–8 mmol/L) in canola oils containing different concentrations of *Teucrium polium* extract (200–1000 ppm) were lower than those (CD = 7–22 mmol/L) in the control sample, independent of the added extract concentration.

Surprisingly, the K_268_ values for the control oil samples and oils loaded with HLRME did not change significantly throughout all heating periods and the frying of fries (Duncan test, Table 3). This suggests that the prolonged high temperature did not accelerate the decomposition of primary oxides into more secondary oxidation products in all studied rapeseed oils. Furthermore, adding HLRME to oils did not significantly affect the amounts of CT and other secondary oxidation products with a characteristic absorption peak at 268 nm during thermal degradation. Therefore, similar K_268_ parameters were measured for non-supplemented (2.426–2.452) and supplemented (2.374–2.455) rapeseed oils before and after the thermo-degradation processes.

On the contrary, Aachary et al. [10] showed that the frying process increased the contents of both CD and CT in canola oils before and after the incorporation of crude canolol extracted from canola meal and phenolic extracts from canola oil deodistillates. However, under heating conditions (185 °C, 5 days), the increases in CD (0.32–1.00 and 0.39–1.15) and CT (0.07–0.22 and 0.09–0.36) were lower for canola oils containing canolol and phenolic extracts, respectively, when compared to control sample (0.41–1.50 and 0.09–0.39 for CD and CT, respectively). The lowest levels of conjugated polyenes throughout the frying period in canola oil with crude canolol confirm its effective inhibition of primary and secondary oxidation product productions during frying.

#### 3.3.6. Total Polar Material

The total polar material (TPM) comprises all the polar degradation products formed during frying, including those from fried fries. As the indicator of the overall quality of oils, TPM provides critical information about the total amount of newly generated compounds having a higher polarity than TAGs and indicates oil deterioration during frying. Therefore, TPM is a suitable parameter for the evaluation of frying oil stability [10,26,53].

The effect of HLRME on the TPM values of heating rapeseed oils during 24 h is presented in Table 3. The increases in TPM results were seen in all oil samples during prolonged heating time (TMP = 13.0–25.5% and 11.5–23.5% for non-supplemented and supplemented rapeseed oils). It is noteworthy that, after the end of experiments, TPM (23.5%) in rapeseed oil fortified with HLRME did not exceed the maximum level permitted in frying oil, representing 25% of TPM, according to regulations in many European countries. In contrast, rapeseed oil without HLRME at the end of the thermo-degradation processes and frying 4 portions of fries had the highest TPM content (25.5%), which is above the legal limits. This finding is consistent with the results for other oxidative parameters (PV, *p*-AnV, TOTOX, INTOX, AV, K_232_), indicating that HLRME could effectively prolong the life of rapeseed oil used for preparation of French fries.

Other authors found similar efficacies of various phenolic extracts in reducing the total polar compounds in different vegetable oils and inhibiting their degradation during frying processes [7,16,20,26,51,53].

Correspondingly, a study by Moufakkir et al. [20] reported that two doses (800 and 1000 ppm) of rosemary extract significantly decreased total polar compounds in soybean oils after 10 h of frying breaded shrimp (TPM = 33, 30, and 25% for soybean oils without and with 800 and 1000 ppm of rosemary extract, respectively). Moreover, the TPM value (22%) of soybean oil containing phenolic extract from *Camellia oleifera* seed cake was significantly lower than the total polar components (29%) in the control sample after all frying cycles [26]. Nevertheless, after 30 h of frying French fries, the non-supplemented and supplemented high-oleic canola oils with 40 and 200 ppm of rosemary extract exceeded the limit of 25% TPM [7]. However, adding canolol-enriched extract reduced TPM levels to 13.2, 10.5, and 10.2% in heated canola oils with 200, 500, and 750 mg/kg of canolol extract.

### 3.4. Effect of Hydrolyzed and Lyophilized Rapeseed Meal Extract and Heating Oil Time on Antioxidant Properties of French Fries

It is well known that oils from the frying media become part of the fried French fries. Thus, bioactive compounds from various sources, such as those naturally present in potatoes and rapeseed oil, incorporated into oil after the addition of HLRME, as well as the different products of their physical and chemical interactions occurring during frying processes as a result of Maillard reaction, Strecker degradation, hydrolysis of esters, and glycosides of antioxidants and oxidation of phenolic antioxidants to quinones and their polymers, affected the antioxidant features of the fried French fries [4,13,25].

The effects of frying medium types (rapeseed oils without and with HLRME) and heating times on the TPC and AA of fried French fries are shown in Table 5.

Evidently, fried French fries in rapeseed oils without and with HLRME exhibited significantly higher amounts of total phenolics and antioxidant potential determined by FC, ABTS, DPPH, and FRAP, respectively, than the frying media used for their preparation (Table 2 and Table 5). These results confirm that the antioxidant properties of fried fries seem to be a sum of antioxidants present in raw potato samples, oils absorbed on their surfaces during frying, active extract added to oils, and new antioxidant compounds generated as products associated with various reactions during the thermal processing of protein-containing foods.

It can be noted that pan-frying frozen fries in oil fortified with HLRME resulted in the preparation of French fries richer in antioxidants compared to those fried in non-supplemented rapeseed oil. All these results indicate that the natural antioxidant compounds present in HLRME improve the quality and antioxidant potential of both heating oil and fried fries (Table 2 and Table 5). However, a gradual and significant decrease in the total amount of phenolics and antioxidant potential in all French fries samples with extended oil heating times was observed (Table 5, Duncan test). Only the Duncan test revealed an insignificant effect of the heating time of fortified rapeseed oil on TPC in French fries (Table 5).

Interestingly, after each 8 h interval of heating, French fries had somewhat higher FRAP (100.25–144.49 and 97.62–123.61 μmol TE/100 g for heating in supplemented and non-supplemented oils, respectively) and ABTS (1128.50–1424.31 and 883.75–1307.82 μmol TE/100 g for heating in supplemented and non-supplemented oils, respectively), whereas DPPH (194.05–447.60 μmol TE/100 g) and TPC (34.97–35.52 mg SAE/100 g) in fries samples after frying in oil with HLRME were about two times higher than those prepared in non-fortified oils (DPPH = 74.16–247.54 and TPC = 19.04–23.51 mg SAE/100 g). This can be explained by the higher retention of antioxidants with the ability to convert the steady radical DPPH to the yellow-colored DPPH-H in the fortified rapeseed oil and their better survival inside the fried fries, which is possibly a result of the high presence of polyphenols. Moreover, each 8 h interval of heating caused a lower decrease in TPC (4.7–7.0%) and ABTS (11.0–29.5%) values for French fries frying in rapeseed oil enriched with HLRME in comparison with control refined rapeseed oil (17.3–33.0% and 8.0–37.8%, for TPC and ABTS results, respectively). On the contrary, the FRAP (7.9–36.1%) of French fries prepared in oil with HLRME decreased faster after each 8-hour heating session than the FRAP (0.7–21.5%) of fries fried in rapeseed oil without active ingredients. However, similar DPPH changes in French fries fried in supplemented (39.2–73.6%) and non-supplemented (11.0–73.3%) oils were observed (Table 5). This can be explained by the fact that the antioxidants naturally present in fries and oils absorbed into them (phenolic compounds, tocopherols, β-carotenes, and vitamins) together with those in added HLRME due to synergistic antioxidant effect among them had a strong inhibitory impact on the degradation of total phenols and other hydrophilic and lipophilic antioxidants capable to scavenging ABTS cation radicals. Furthermore, interactions between lower free radical amounts in degraded oils in fries and naturally present free radical scavengers in raw potatoes and incorporated HLRME could not be excluded. In contrast, the bioactive compounds from HLRME added to rapeseed oil did not protect French fries’ redox-active components (electron-donating antioxidants) before they were degraded in heated conditions.

Similarly, French fries pan-fried in sunflower, olive, and palm oils fortified with olive leaf extract at levels of 120 and 240 mg total polyphenols per kg of each oil had a higher DPPH radical scavenging activity (IC_50_ = 15.2–174 mg) than the respective ones fried in control oils without olive leaf extract (IC_50_ = 54.0–454 mg) [11].

Moreover, the slower degradation of individual antioxidants, including tocopherols, oleuropein, and chlorogenic acid in French fries fried in vegetable oils enriched with enzymatically prepared octadecyl chlorogenate from rowanberry extract, olive leaf extract, and, olive mill waste water, respectively, compared to fries prepared in those oils without phenolic extracts, were observed by Aladedunye et al. [3], Chiou et al. [19], and Sordini et al. [15]. The total amounts of tocopherols in the French fries deep-fried in rapeseed oil without and with natural extract and lipophilic extract from rowanberry during 8 hours of frying process decreased from 70, 72, and 76 μg/g fries to 10, 20, and 22 μg/g fries, respectively [3]. However, oleuropein was present in French fries (0.22–0.29 mg/100 g), even after the eighth frying session in olive, sunflower, and soybean oils enriched with an oleuropein-rich olive leaf extract [19].

In addition, Sordini et al. [15] found that a higher concentration of phenolic extract from olive mill wastewater added to refined olive oil caused a lower loss of potato phenols during the frying simulation process. The first loss in the chlorogenic acid concentration from raw frozen, pre-fried potatoes to cooked French fries was 52% and 32–43% in non-supplemented and supplemented olive oils, respectively. This clearly indicated that the decrease was influenced by the oil’s phenolic antioxidant composition, confirming that the high polyphenol concentrations in an extract from olive mill wastewater guaranteed overall protection of the fried food and the oil itself against oxidative degradation.

### 3.5. Unsupervised Multivariate Techniques

In this study, two unsupervised multivariate techniques, principal component analysis (PCA) and hierarchical cluster analysis (HCA), were applied for multivariate associations between oxidative status and antioxidant features of non-supplemented and supplemented rapeseed oils before and after the thermo-degradation processes and frying of French fries.

The PCA was used to reveal the effects of heating time (0–24 h) and frying medium (refined rapeseed oils without and with HLRME) on the TPC and AA of oils and fried French fries. However, HCA as a clustering method was chosen to explore the organization of all oil samples (without and with HLRME, before and after frying) in groups and among groups, depicting a hierarchy based on their AA determined by ABTS, DPPH, FRAP, the total amount of polyphenols analyzed by FC assay, and oxidative parameters (PV, *p*-AnV, TOTOX, INTOX, AV, K_232_, K_268_, TPM).

#### 3.5.1. Principal Component Analysis

**Figure 3:**
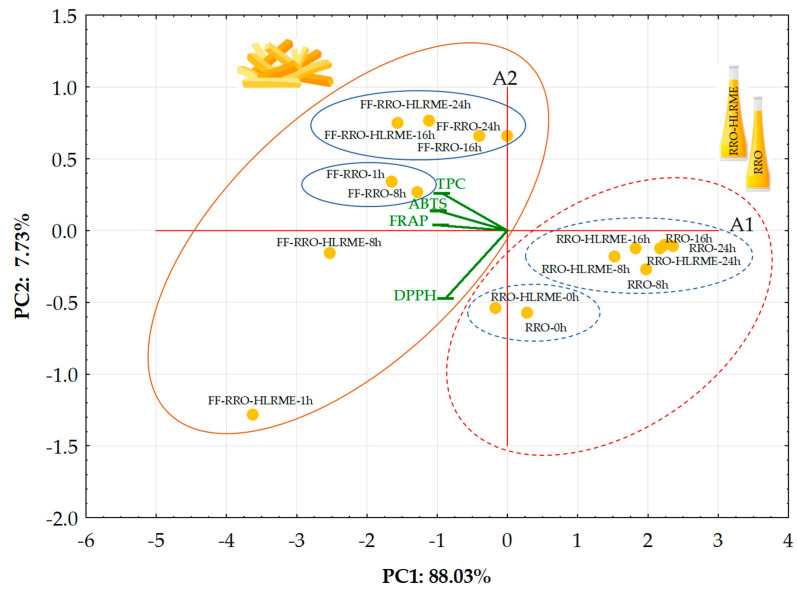
Biplot of scores and loadings of data obtained for antioxidant properties of fried French fries and non-supplemented and supplemented rapeseed oils utilized for their preparation after different heating times. Big circles separate the frying media (dashed line) from fried French fries in them (solid line).

A bi-plot of PC2 versus PC1 determining the most important variables that explain the relationships between non-supplemented (RRO) and supplemented (RRO-HLRME) rapeseed oils and French fries (FF) fried after different oil heating cycles and identifying any group pattern is presented in Figure 3.

The results of PCA revealed that the first two principal components, PC1 and PC2, accounted for 88.03% and 7.73% of the variance, respectively (95.76% in total). PC1 was negatively correlated with all variables: TPC (−0.935), ABTS (−0.982), DPPH (−0.877), and FRAP (−0.956), while PC2 was highly negatively linked to DPPH (−0.470).

The biplot shows that PC1 separated the studied samples into two general groups: the negative sector, including French fries fried in two types of oils (without and with HLRME) and the positive sector, consisting of only oils heated at different times (Figure 3). It is noteworthy that the studied samples fell into four distinct groups, from which two French fries samples fried in enriched oil in the first heating cycles were separated (Figure 3).

The French fries fried in refined rapeseed oil supplemented with HLRME at the shortest time (1 h) with the longest distance from other investigated samples revealed the highest TPC and AA determined by three analytical methods. However, before the frying process, the fortified rapeseed oil (RRO-HLRME-0h) and refined rapeseed oil without HLRME (RRO-0h) created an evidently distinct cluster due to the above two times lower ABTS and DPPH values. Interestingly, oils enriched with HLRME after more extended times of thermo-degradation processes (8–24 h) were grouped together with the heated rapeseed oils without HLRME. These oils generally had similar ABTS (230.90–297.84 and 181.80–238.07 μmol TE/100 g for supplemented and non-supplemented oils, respectively) and TPC (2.70–3.15 and 2.00–2.50 mg SAE/100 g for supplemented and non-supplemented oils, respectively), whereas DPPH (36.02–72.26 μmol TE/100 g) and FRAP (17.80–62.35 μmol TE/100 g) results for fortified oils were about two times higher than those for non-fortified oils (DPPH = 20.36–71.95 and FRAP = 9.98–27.71 μmol TE/100 g).

On the other hand, the sample of French fries fried in fortified rapeseed oil after 8 h of heating separated from a group of two fries samples prepared in non-fortified oils heated for shorter (1 h) and the same times (8 h). The slight distance from these three samples of French fries can be explained by approximately two times lower DPPH (247.54–278.19 μmol TE/100 g) and TPC (23.51–28.42 mg SAE/100 g) values for fries fried in non-enrich rapeseed oils than DPPH (447.60 μmol TE/100 g) and TPC (35.52 mg SAE/100 g) in fries fried in rapeseed oil with HLRME after 8 hours of heating. The last cluster contained the French fries fried in refined rapeseed oils without and with HLRME after longer heating times (16 and 24 h), which were characterized by the lowest level of antioxidants. However, the fries prepared in the fortified oil had higher antioxidant properties (TPC = 34.97–35.81 mg SAE/100 g, ABTS = 1128.50–1227.23 μmol TE/100 g, DPPH = 194.05–221.74 μmol TE/100 g, and FRAP = 100.25–123.84 μmol TE/100 g) than the respective ones fried in non-fortified oil (TPC = 19.04–19.56 mg SAE/100 g, ABTS = 883.75–1043.41 μmol TE/100 g, DPPH = 74.16–99.70 μmol TE/100 g, and FRAP = 97.62–112.72 μmol TE/100 g), which caused a slight shift of these two samples to the right side of the PCA graph.

In addition, the PCA biplot graphic provided information about correlations between the determined antioxidant properties of frying media and the fried French fries. The DPPH of the analyzed samples was the variable with negative loadings on PC1 and PC2, while TPC, ABTS, and FRAP were the features with negative loadings on PC1 and positive loadings on PC2. It is noteworthy that FRAP, ABTS, and TPC, being the closer loadings on the PCA biplot, exhibited the highest correlation coefficient values (r = 0.8413–0.9446). However, the lowest positive correlation (r = 0.7202) was observed between DPPH and TPC (the longest distance from those variables).

The results obtained by PCA demonstrate the differences between all examined samples due to the addition of HLRME to refined rapeseed oil and its impact on the antioxidant properties of oils and products during the frying processes.

#### 3.5.2. Hierarchical Cluster Analysis

**Figure 4:**
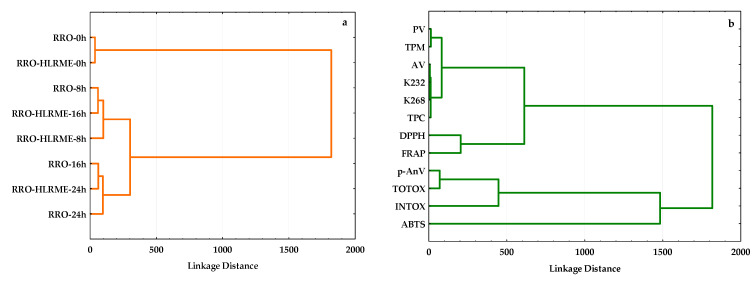
Dendrograms depicting the hierarchical clustering of (**a**) the frying media and (**b**) their antioxidative and oxidative parameters.

HCA was utilized to group all studied frying media, including rapeseed oils without and with HLRME heated at different times based on similarities of their antioxidant potential determined by three analytical methods (ABTS, DPPH, and FRAP), total amounts of phenolic compounds (TPC) and polar compounds (TPM), and oxidative status (PV, *p*-AnV, TOTOX, INTOX, AV, K_232_, and K_268_). Moreover, HCA was performed to reveal relationships between the analyzed antioxidant features and oxidative parameters of the investigated oils and created groups among them. Two-dimensional visions of the similarity and dissimilarity of the studied oil samples and their antioxidant and oxidative parameters are presented on dendrograms. The dendrogram of the frying media is shown in Figure 4a, whereas the correlations between the analyzed variables are illustrated in Figure 4b.

It can be noted that the eight refined rapeseed oils, four supplemented with HLRME and four without any active ingredient treated after various heating times, were classified into two main clusters (Figure 4a). Fresh, refined rapeseed oils without and with HLRME before the heating process belong to the first cluster due to the lowest oxidative parameters and the highest total antioxidant amounts. However, the second cluster comprises two subgroups, each consisting of enriched oil after 8 h longer heating time than control oil without HLRME. Interestingly, rapeseed oil supplemented with HLRME used for frying French fries after 8 h of heating and the non-supplemented and longest heated (24 h) rapeseed oil quite separated from the first and second subgroups, respectively. This can be explained by the fact that eight-hour heating of the enriched oil (RRO-HLRME-8h) resulted in the generation of lower amounts of primary (PV) and secondary (*p*-AnV) oxidation products, free fatty acids (AV), total polar compounds (TPM), and conjugated dienes (K_232_) and trienes (K_268_) with lower degradation of active components (higher values of TPC, ABTS, DPPH, and FRAP) compared to the more significant oxidative changes and disruption of antioxidants occurring in oils without HLRME (RRO-8h) and with HLRME (RRO-HLRME-16h) heated for 8 and 16 h, respectively (Table 2 and Table 3). However, refined rapeseed oil without HLRME (RRO-24h) stood out in the second subgroup due to its lowest antioxidant potential (ABTS = 181.80 μmol TE/100 g, DPPH = 20.36 μmol TE/100 g, and FRAP = 9.98 μmol TE/100 g), polyphenolic content (TPC = 2.00 mg SAE/100 g), and high oxidative state (PV = 17.16 meq O_2_/kg, *p*-AnV = 186.78, TOTOX = 221.1, INTOX = 390.72, AV = 0.278, K_232_ = 2.814, K_268_ = 2.429, TPM = 25.5).

Additionally, an HCA dendrogram of antioxidative and oxidative parameters measured for the fresh and heated rapeseed oils without and with HLRME is represented in Figure 4b. The AA results obtained by FRAP and DPPH methods, total phenolic amounts (TPC), indicators of oxidative status such as contents of conjugated diene (K_232_) and trienes (K_268_), free fatty acids (AV) and hydroperoxides (PV), as well as frying stability (TPM) created one cluster divided into two subgroups. The first subgroup illustrated the influence of the phenolic compounds (TPC) on concentrations of conjugated polyenes (K_232_ and K_268_), free fatty acids (AV), polar components (TPM), and hydroperoxides (PV) formed in oils during thermo-degradation processes. Unexpectedly, DPPH and FRAP assays formed the second subgroup in this cluster. This suggests that the studied oil samples contained antioxidants that could simultaneously reduce iron and quench DPPH radicals based on a similar mechanism of transfer of electrons from the antioxidant to reduce an oxidant for these two analytical methods. However, the second cluster consisted of three oxidative status parameters of the studied oils: amounts of secondary oxidation products (*p*-AnV), TOTOX, and INTOX indicators, whereas hydrophilic and lipophilic antioxidants in oil samples determined by the ABTS decolorization assay separated from this group (Figure 4b). This fact confirms that the secondary oxidation products significantly affected the total oxidation of TAGs in all rapeseed oils heated at different times to prepare fried French fries. Moreover, the indirect ABTS assay provided more information on the antioxidant features of the studied rapeseed oils during advanced oxidation processes than the DPPH and FRAP methods.

#### 3.5.3. Color Map of Correlations

**Figure 5:**
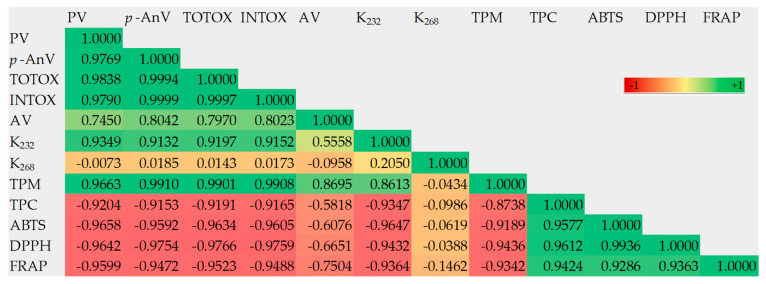
Color matrix of correlations between analyzed antioxidant features and oxidative status parameters of non-supplemented and supplemented rapeseed oils utilized for frying French fries after different heating times.

The results of the correlation analysis between the studied variables of the fresh and heated rapeseed oils without and with HLRME are presented as a color matrix in Figure 5.

The degree of linear relationship between the studied variables was determined using regression analysis and calculating the Pearson correlation coefficient (r). The color matrix illustrated the positive significant relationships (r = 0.7450–0.9999, *p* < 0.05) between oxidative parameters such as PV, *p*-AnV, TOTOX, INTOX, K_232_, and AV (except AV–K_232_, r = 0.5558, *p* = 0.1526). Meanwhile, the highest correlation coefficients were observed between the *p*-AnV, INTOX, and TOTOX indexes, creating a separate cluster in the dendrogram (Figure 4b). A somewhat higher degree of linear association for *p*-AnV and INTOX indicator (r = 0.9999) than this for *p*-AnV and TOTOX indicator (r = 0.9994) suggests the more substantial effect of secondary oxidation products on an integral oxidation state of the tested oils than in their overall oxidation state. In addition, positive significant correlations (r = 0.8613–0.9910, *p* < 0.05) were identified between these oxidative parameters and the total amount of polar compounds (TPM) generated during thermal degradation processes. On the contrary, the effect of conjugated trienes (K_268_) in the investigated oils on their oxidative states (r = −0.0073–0.2050, *p* = 0.6263–0.9863), TPM (r = −0.0434, *p* = 0.9188), and antioxidant properties (r = −0.0388–−0.1462, *p* = 0.7298–0.9272) was insignificant. This can be explained by the fact that CT formed during the heating process in refined rapeseed oils without and with HLRME contributed insignificantly to their oxidative stability and antioxidant properties. Moreover, all oxidative parameters (except K_268_ and AV) and polar components (TPM) present in all studied oils were significantly negatively correlated with the total concentration of phenolic compounds (r = −0.8738–−0.9347, *p* = 0.000662–0.00456) and AA determined by three analytical methods (r = −0.9189–−0.9766, *p* = 0.000031–0.00126). It is worth emphasizing that there were insignificant but linear correlations (r = −0.5818–−0.6651, *p* = 0.00719–0.1303) between free fatty acids (AV) in all oils and their antioxidant features determined by FC, ABTS, and DPPH tests. This suggests that the acidity index decreased with increasing antioxidants in oil samples.

Evidently, the high r values (0.9286–0.9936, *p* > 0.05) indicated strong relationships between the singlet electron transfer (SET)-based FRAP method and the scavenging activity of antioxidants present in the investigated oils toward stable ABTS cation radicals and DPPH radicals. Therefore, all oil samples without and with HLRME before and after frying French fries contained antioxidant compounds able to reduce colorless ferric-tripyridyltriazine (Fe(III)-TPTZ) to an intense blue color ferrous-tripyridyltriazine complex (Fe(II)-TPTZ) and scavenge ABTS and DPPH radicals. In addition, all phenolics (TPC) in these oils contributed significantly positively to antioxidant activities measured using three analytical methods; thus, correlation coefficients for relationships between TPC and ABTS, DPPH, and FRAP varied from 0.9424 to 0.9612.

Further research is needed to better understand the mechanisms of new compound synthesis during the heating of fortified rapeseed oils and frying of French fries, as well as the interactions between them and naturally present bioactive compounds.

## 4. Conclusions

Processing oil and especially its oxidative state and antioxidant properties are the most important factors in frying safe French fries. For this reason, the presented results can help develop a safety management system for producing healthy oils, which can be utilized in frying French fries. The addition of rapeseed meal hydrolysate after acid hydrolysis and lyophilization (HLRME) rich in antioxidants to refined rapeseed oil enhanced its antioxidant properties, as analyzed by ABTS, DPPH, FRAP, and FC analytical methods, and oxidative status by preventing an increase in the oxidative parameters (PV, *p*-AnV, TOTOX, INTOX, and CD) and the formation of total polar components (TPM) during thermo-degradation processes and frying French fries. Furthermore, rapeseed oil antioxidant features before and after fortification with HLRME affected the antioxidant potential of French fries prepared in these two frying media at different intervals. The enrichment of rapeseed oil with HLRME significantly improved the antioxidant quality of fried French fries compared to those prepared in non-fortified oils. Incorporating phenolic compounds from rapeseed by-products into rapeseed oil opens new possibilities in innovative foods, allowing their integral valorization and enhancing the oil industry’s profitability. However, further research is needed to investigate various concentrations of added HLRME with antioxidant potential that can effectively inhibit advanced oxidation processes in refined rapeseed oil, reflecting its quality during thermal oxidation as it will condition the quality of the fried food.

## Figures and Tables

**Table 1:** Total phenolic content and antioxidant activity of crude rapeseed meal extract and after alkaline and acid hydrolysis.

Rapeseed Meal Extract	Antioxidant Features			
	TPC * ± SD (mg SAE/g)	ABTS * ± SD (µmol TE/g)	DPPH * ± SD (µmol TE/g)	FRAP * ± SD (µmol TE/g)
Crude	12.20 ± 0.48 ^a^	846.20 ± 19.91 ^a^	126.40 ± 1.61 ^a^	508.61 ± 17.20 ^a^
After alkaline hydrolysis	18.42 ± 0.10 ^b^	4910.54 ± 186.41 ^c^	981.09 ± 45.07 ^b^	586.47 ± 26.62 ^b^
After acid hydrolysis	24.80 ± 0.87 ^c^	3880.81 ± 150.26 ^b^	132.03 ± 6.17 ^a^	650.24 ± 13.48 ^c^

* *n* = 3; SD—standard deviation; different letters (^a–c^) within the same column indicate significant differences between antioxidant properties of rapeseed meal extracts (one-way ANOVA and Duncan test, *p* < 0.05).

**Table 2:** Changes in total phenolic content and antioxidant activity of refined rapeseed oil without and with hydrolyzed and lyophilized rapeseed meal extract (HLRME) during different heating times before frying French fries.

Heating Time (h)	Antioxidant Features of Frying Medium			
	TPC * ± SD (mg SAE/100 g)	ABTS * ± SD (µmol TE/100 g)	DPPH * ± SD (µmol TE/100 g)	FRAP * ± SD (µmol TE/100 g)
Refined Rapeseed Oil without HLRME				
0	6.04 ± 0.02 ^f^	733.25 ± 18.36 ^d^	235.34 ± 5.88 ^f^	91.72 ± 2.99 ^f^
8	2.50 ± 0.06 ^b^	238.07 ± 4.81 ^b^	71.95 ± 2.72 ^e^	27.71 ± 1.42 ^c^
16	2.43 ± 0.11 ^b^	184.34 ± 5.34 ^a^	25.25 ± 1.71 ^b^	18.41 ± 1.11 ^b^
24	2.00 ± 0.10 ^a^	181.80 ± 8.05 ^a^	20.36 ± 1.19 ^a^	9.98 ± 0.46 ^a^
Refined Rapeseed Oil with HLRME				
0	8.57 ± 0.09 ^g^	746.24 ± 14.02 ^d^	256.33 ± 7.64 ^g^	119.66 ± 4.21 ^g^
8	3.15 ± 0.06 ^e^	297.84 ± 10.67 ^c^	72.26 ± 3.46 ^e^	62.35 ± 5.17 ^e^
16	2.81 ± 0.04 ^d^	229.01 ± 5.93 ^b^	46.40 ± 0.43 ^d^	47.83 ± 2.26 ^d^
24	2.70 ± 0.07 ^c^	230.90 ± 4.63 ^b^	36.02 ± 1.46 ^c^	17.80 ± 0.82 ^b^

* *n* = 3; SD—standard deviation; different letters (^a–g^) within the same column indicate significant differences between total phenolic content and antioxidant activity of non-supplemented and supplemented rapeseed oils heating for 0 to 24 h (one-way ANOVA and Duncan test, *p* < 0.05).

**Table 3:** Evolution of primary (PV) and secondary (*p*-AnV) oxidation products, TOTOX and INTOX indicators, free fatty acids (AV), conjugated diene (K_232_), conjugated triene (K_268_), and total polar material (TPM) in refined rapeseed oils without and with hydrolyzed and lyophilized rapeseed meal extract (HLRME) during different heating times.

Heating Time (h)		Oxidation Parameters						
	PV * ± SD (meq O_2_/kg)	*p*-AnV * ± SD	TOTOX	INTOX	AV * ± SD (mg NaOH/g)	K_232_ * ± SD	K_268_ * ± SD	TPM (%)
Refined Rapeseed Oil without HLRME								
0	0.18 ± 0.04 ^a^	1.37 ± 0.03 ^a^	1.73	2.92	0.022 ± 0.005 ^a^	0.562 ± 0.008 ^a^	2.426 ± 0.028 ^a^	
8	14.15 ± 0.14 ^c^	111.41 ± 1.28 ^c^	139.71	236.97	0.036 ± 0.002 ^b^	2.789 ± 0.034 ^d^	2.452 ± 0.036 ^a^	13.0
16	14.23 ± 0.81 ^c^	148.81 ± 2.11 ^e^	177.27	311.85	0.141 ± 0.007 ^d^	2.770 ± 0.051 ^d^	2.441 ± 0.040 ^a^	19.0
24	17.16 ± 0.59 ^e^	186.78 ± 2.01 ^g^	221.10	390.72	0.278 ± 0.013 ^f^	2.814 ± 0.083 ^d^	2.429 ± 0.062 ^a^	25.5
Refined Rapeseed Oil with HLRME								
0	0.36 ± 0.03 ^a^	1.08 ± 0.03 ^a^	1.80	2.52	0.015 ± 0.01 ^a^	0.498 ± 0.009 ^a^	2.425 ± 0.051 ^a^	
8	8.73 ± 0.18 ^b^	104.88 ± 2.24 ^b^	122.34	218.49	0.026 ± 0.002 ^a,b^	2.036 ± 0.008 ^b^	2.455 ± 0.047 ^a^	11.5
16	13.99 ± 0.18 ^c^	131.47 ± 0.30 ^d^	159.45	276.93	0.103 ± 0.010 ^c^	2.100 ± 0.048 ^b,c^	2.374 ± 0.029 ^a^	17.5
24	16.40 ± 0.48 ^d^	163.38 ± 3.18 ^f^	196.18	343.16	0.250 ± 0.001 ^e^	2.144 ± 0.058 ^c^	2.434 ± 0.082 ^a^	23.5

* *n* = 3; SD—standard deviation; different letters (^a–g^) within the same column indicate significant differences between oxidative parameters of non-supplemented and supplemented rapeseed oils heating for 0 to 24 h (one-way ANOVA and Duncan test, *p* < 0.05).

**Table 4:** Regression analysis and adjusted determination coefficients for the dependent variables: PV, *p*-AnV, TOTOX, and INTOX indicators of rapeseed oils without and with HLRME evaluated during the thermo-degradation processes.

Dependent Variable	Oil Sample	Regression *		R^2^
		Intercept (β_0_)	Slope (β_1_)	
PV	RRO	3.78	0.64	0.75
	RRO-HLRME	1.86	0.67	0.94
*p*-AnV	RRO	23.05	7.42	0.92
	RRO-HLRME	23.18	6.42	0.89
TOTOX	RRO	30.60	8.70	0.90
	RRO-HLRME	26.91	7.75	0.90
INTOX	RRO	49.87	15.48	0.91
	RRO-HLRME	48.22	13.51	0.89

* Regression equation: Y = β_0_ + β_1_X, where Y—dependent variable (PV, *p*-AnV, TOTOX, and INTOX); β_0_—intercept; β_1_—slope; X—independent variable (heating time); R^2^—adjusted determination coefficient.

**Table 5:** Effect of frying medium and thermal processing time on total phenolic content and antioxidant activity of fried French fries.

Heating Time (h)	Antioxidant Features of Fried French Fries			
	TPC * ± SD	ABTS * ± SD	DPPH * ± SD	FRAP * ± SD
	(mg SAE/100 g)	(µmol TE/100 g)	(µmol TE/100 g)	(µmol TE/100 g)
Refined Rapeseed Oil without HLRME				
1	28.42 ± 1.32 ^c^	1421.19 ± 45.27 ^f^	278.19 ± 8.17 ^f^	124.42 ± 7.13 ^c^
8	23.51 ± 0.85 ^b,c^	1307.82 ± 56.00 ^e^	247.54 ± 11.86 ^e^	123.61 ± 5.86 ^c^
16	19.56 ± 0.98 ^a,b^	1043.41 ± 32.28 ^b^	99.70 ± 3.36 ^b^	112.72 ± 7.45 ^b^
24	19.04 ± 0.49 ^a^	883.75 ± 39.55 ^a^	74.16 ± 5.19 ^a^	97.62 ± 3.05 ^a^
Refined Rapeseed Oil with HLRME				
1	37.57 ± 1.70 ^d^	1600.60 ± 27.17 ^g^	735.65 ± 10.79 ^h^	156.96 ± 4.99 ^e^
8	35.52 ± 0.63 ^d^	1424.31 ± 49.11 ^f^	447.60 ± 12.44 ^g^	144.49 ± 6.84 ^d^
16	35.81 ± 1.01 ^d^	1227.23 ± 23.67 ^d^	221.74 ± 8.51 ^d^	123.84 ± 2.32 ^c^
24	34.97 ± 1.43 ^d^	1128.50 ± 43.47 ^c^	194.05 ± 5.32 ^c^	100.25 ± 2.63 ^a^

* *n* = 3; SD—standard deviation; different letters (^a–h^) within the same column indicate significant differences between antioxidant features of fried French fries in non-supplemented and supplemented rapeseed oils heated for 1 to 24 h (one-way ANOVA and Duncan test, *p* < 0.05).

## Data Availability

The original contributions presented in the study are included in the article. Further inquiries can be directed to the corresponding author.
